# Assessing the Use of 3D-Model Prostheses in White Storks: A Promising Method in Rehabilitation of Injured Wildlife

**DOI:** 10.3390/biology14030265

**Published:** 2025-03-05

**Authors:** Rusko Petrov, Catarina Quinteira, Stefka Dimitrova

**Affiliations:** 1 Department of General Animal Husbandry, Faculty of Veterinary Medicine, Trakia University—Stara Zagora, 6000 Stara Zagora, Bulgaria; 2 Green Balkans—Stara Zagora NGO, 6000 Stara Zagora, Bulgaria; 3 Department of Agricultural and Veterinary Sciences, University of Trás-os-Montes and Alto Douro, Quinta de Prados, 5000-801 Vila Real, Portugal

**Keywords:** conservation medicine, orthopedic prostheses, 3D printing, GPS tracking, *Ciconia ciconia*, wildlife rehabilitation

## Abstract

Wildlife Rehabilitation Centres emerged with the purpose of recovering individuals, as a tool for environmental education and monitoring the balance of ecosystems. The White Stork (*Ciconia ciconia*) is one of the many species that are admitted to rehabilitation centres all around the world, due to traumatic amputations. This work presents the development of 3D-printed orthopedic prostheses aimed at partially restoring biomechanical function and enabling the reintegration of amputated birds into their natural habitat. Conducted at the Green Balkans Wildlife Rehabilitation and Breeding Center in Bulgaria, three prosthetic prototypes were created using epoxy resin, polylactic acid (PLA), and polyamide, based on detailed anatomical measurements. The process involved 3D Computer-Aided Design (CAD), biomechanical analysis, and performance evaluation, focusing on locomotion, feeding, and flight. Results showed improved prosthetic efficacy, with birds adapting within 1–5 days, resuming normal behaviours, and regaining flight. Of the 12 birds analyzed, 3 were released into the wild, with 1 tracked via GPS, marking the first documented case of an amputated bird with a prosthesis monitored post-release, covering over 470 km in 15 days. This study highlights the potential of 3D printing in conservation medicine, offering alternatives to euthanasia and open new perspectives in the global context of biodiversity preservation.

## Simple Summary

The white stork is often admitted to rehabilitation centres with severe injuries, including traumatic amputations. The purpose of this research was to design and evaluate 3D-printed orthopedics prostheses to restore partial mobility and improve survival chances. Conducted at the Green Balkans Wildlife Rehabilitation Center in Bulgaria, the research involved designing three prosthetic models using advanced materials and biomechanical analysis. The study evaluated their effectiveness in allowing birds to walk, feed, and fly again. Results showed that storks adapted to the prostheses within days, with some successfully returning to the wild. One bird was monitored via GPS after its release, marking the first documented case of a prosthetically equipped bird being tracked in nature. It travelled over 470 km in 15 days, demonstrating the success of this innovative approach. This research highlights the potential of 3D printing in wildlife conservation, providing viable alternatives to euthanasia and supporting species rehabilitation. Future studies may explore applications for other species, contributing to biodiversity preservation.

## 1. Introduction

Wildlife is a prime biological monitor of environmental condition, reflecting the state of balance and health in ecosystems, including cities [1]. Monitoring such animals is vital for ecological and conservation science because it helps in determining and adjusting drivers of declines in wildlife populations [2]. Wildlife Rehabilitation Centres were established to aid in the rehabilitation of a single animal, but also as tools of environmental education and ecosystem monitoring [3]. The centres are ecological study hubs, which give us additional information about wildlife health and population dynamics [2].

Some of these animals suffer permanent injuries, such as traumatic amputations, which make them non-viable in the wild [4]. New technologies in veterinary orthopedics, such as the development of 3D-printed prosthetics, offer potential interventions for enhancing the quality of life and potential reintegration into the wild of these animals [5].

Three-dimensional (3D) printing has revolutionized the production of prosthetics in veterinary medicine with tailored interventions for a large variety of species. Its precision and adaptability allow the creation of light, anatomically correct prosthetics using materials like polylactic acid (PLA), polyamide, and resins. In avian medicine, 3D printing has been used with positive effects in the repair of damaged beaks and limbs, greatly improving the quality of life in the birds involved. Some excellent examples are toucan and eagle prosthetic beaks, enabling them to eat and preen independently [6,7]. The same has been true for limb prosthetics, but challenges continue to exist in mimicking natural movement and equipment longevity [8]. These early signs show the potential of 3D printing in wildlife rehabilitation, particularly in species with specific biomechanical needs.

The White Stork (*Ciconia ciconia*) is a fascinating case study in this regard. Frequently encountered in rehabilitation centres across Europe for traumatic injuries like limb amputations resulting from collisions, electrocutions, and entanglements with agricultural by-products [9,10], the species poses specific challenges and opportunities for prosthetic use. As a migratory bird with specialized biomechanical needs, partial limb function must be restored to facilitate successful reintroduction into the wild.

This study involves the production and application of 3D-printed orthopedic prostheses in White Storks with pelvic limb amputations. The main goal is the restoration of some biomechanical functionality to support locomotion, feeding, and flight, and the exploration of the potential for successful release to natural habitats. The project was carried out at the Green Balkans Wildlife Rehabilitation and Breeding Center in Bulgaria, where three prototyped prosthetics—produced from epoxy resin, polylactic acid (PLA), and polyamide—were also designed using accurate anatomical measurements.

New methods were used, such as Computer-Aided Design (CAD) to model 3D, biomechanical analysis, and performance tests with emphasis on key behaviours such as locomotion and flight. Preliminary results indicated that birds adapted to the prosthetics within 1–5 days, resuming natural activities. Of the 12 individuals fitted with prosthetics, three were successfully reintroduced into the wild, with one monitored via GPS, marking the first documented case of a bird equipped with an orthopedic prosthesis and tracked post-release. Through the analysis of GPS data, we observed that this stork covered over 470 km in 15 days.

This new approach emphasizes the potential of 3D printing to be applied in conservation medicine and presents alternatives to euthanasia for severely injured wildlife. Future research aims to advance prosthetic design and expand its application to other birds of prey species, further broadening the scope of this technology towards preserving biodiversity.

## 2. Materials

Green Balkans’ Wildlife Rehabilitation and Breeding Centre (Stara Zagora, Bulgaria) is the only facility of this kind functioning in the country. Since 2014, the Wildlife Rehabilitation and Breeding Centre and Trakia University (Stara Zagora, Bulgaria) collaborated to find an option for the numerous birds who arrive at the wildlife rescue centre with damaged beaks and legs. These birds were destined to remain in captivity or be euthanized since they cannot be effectively rehabilitated, so a team of veterinarians has led an innovative study that employs technology to advance rehabilitation of these birds.

**Figure 1:**
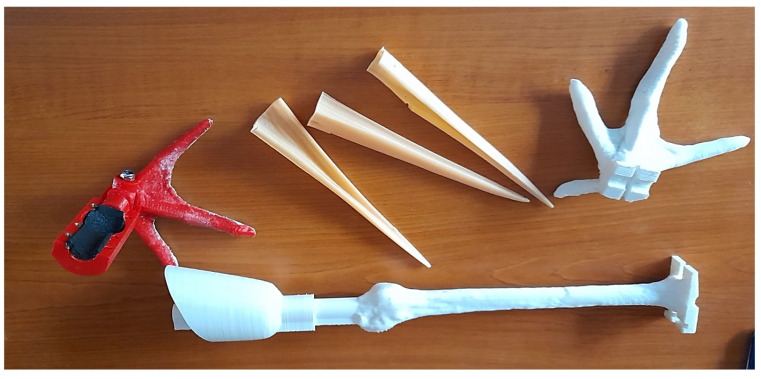
Prototypes of legs and beaks prosthetics developed by the team of veterinarians and 3D AMS.

From 2014 to 2020, the team developed a prosthetic leg made of epoxy resin (Figure 1). In total, six storks were implanted with this prosthesis and of these, only one was returned to the wild in October 2015. The other five remained in the recovery centre and experienced some complications: the surgical prosthesis either fell out because the hollow bones could not support the connection, or the implanted components caused small wounds at the attachment site.

In 2021, Green Balkans’ veterinarian team started to collaborate with a 3D printing company, 3D AMS (Stara Zagora, Bulgaria) to develop and 3D-print some prototypes of leg and beak prosthetics (Figure 1).

**Figure 2:**
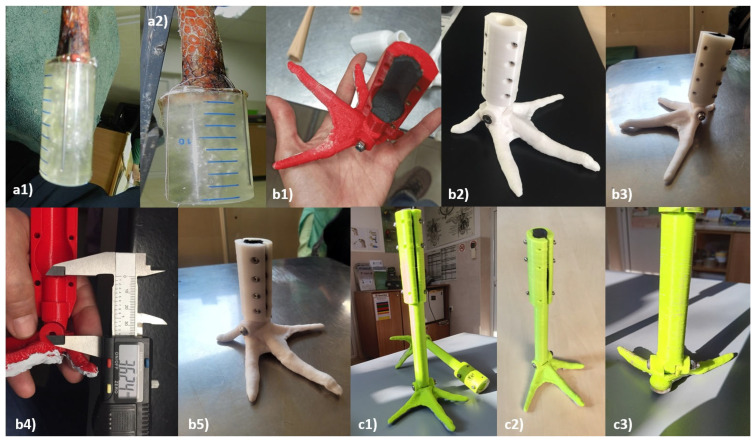
(**a1**,**a2**) P1—Prosthetic leg made of epoxy resin and a syringe; (**b1**–**b5**) P2—Different angles of the second model made with PLA, the red and white version; (**c1**–**c3**) P3—The biggest prosthetic model made with polyamide.

This study has been conducted with white storks of various ages, genders, and types of amputation. Table 1 and Figure 2 provide a comprehensive overview of the different prosthetic leg models developed, allowing for a clearer analysis of their materials, manufacturing methods, advantages, and limitations.

## 3. Methods

In cases where birds were admitted with serious fractures, burns, or any other injury that could not be treated, amputation surgery on the affected limb was planned.

Prior to the surgery, the anesthetic plan was adjusted based on the bird’s weight. Ketamine and isoflurane are used usually as premedication of anesthesia for maintenance in storks. After the stork was anesthetized, the amputation location was evaluated to determine how much skin should be left to cover the bone, and then surgical asepsis was performed with povidone iodine.

After the preparation of the skin around the amputation site, the vessels were ligated proximally and distally to prevent bleeding and field contamination. The necrotic region of the bone was then removed using a surgical saw, and the skin was sutured. In the postoperative period, a soft dry dressing was applied and examined every 3–4 days. In cases where there was not enough skin to suture, pine tar was used to create a bandage to seal the bone and prevent infections.

Since it took some time after surgery to place the orthopedic prosthesis, it was recommended to apply a soft bandage to the foot of the other limb to prevent the development of pododermatitis.

**Figure 3:**
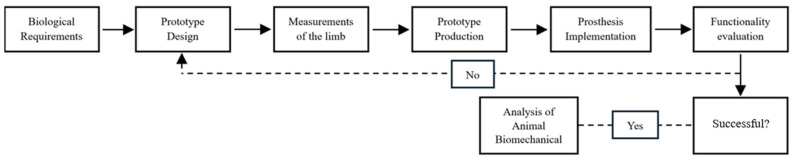
Multi-phase process to develop the prosthesis.

To develop the prosthesis as efficiently as possible, several prototypes were made through a process that included a multi-phase process that is described below (Figure 3).

The phases included in the methodology are described as follows:

Phase 1—Biological requirements: Planning the design of the prototype to better adapt to the needs, activities and behaviours of the animal.

**Figure 4:**
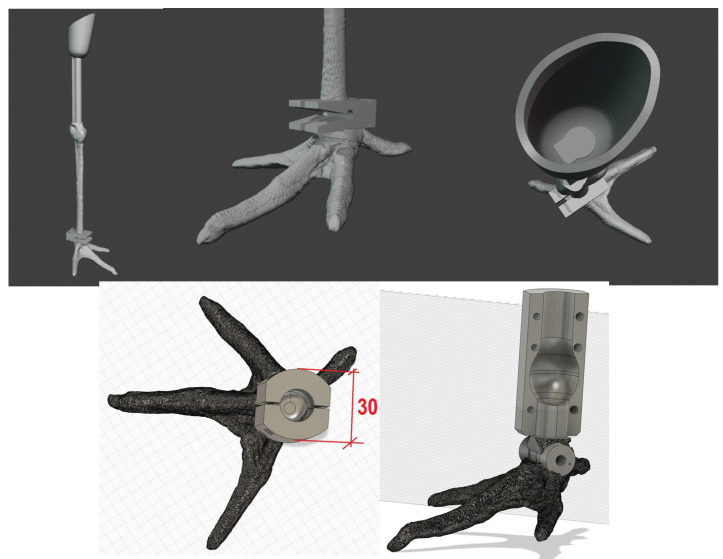
Three-dimensional renderings of the design of the prosthesis.

Phase 2—Using 3D software—AutoCAD 24.2, the prosthesis design was planned by employing a pelvic limb from a white stork cadaver as a reference to replicate the natural structure of the stork’s pelvic limb, including all its digits (Figure 4).

The prostheses were mostly static, meaning they did not allow mimicking joint movements, but they did contain a small hinge at the junction of the tarsometatarsus and the foot, which allowed a flexion movement of around 30°.

**Figure 5:**
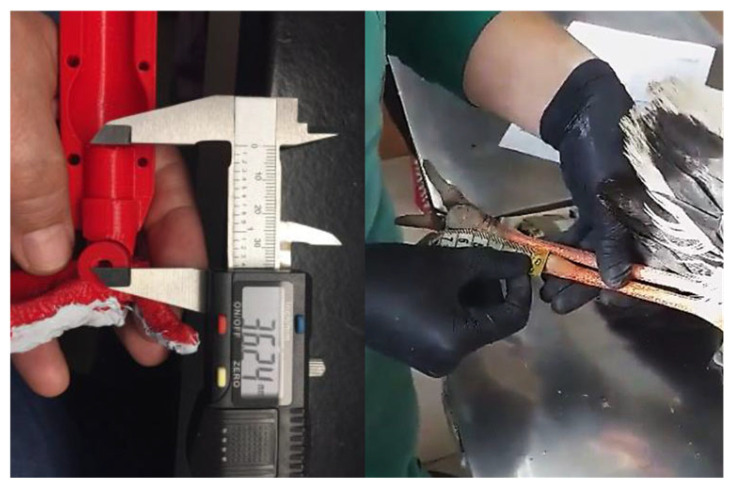
Measurements of the healthy limb.

Phase 3—Measurements of the limb: Using a measuring tape or calliper, the lengths of the prosthesis were determined in relation to the healthy leg (Figure 5). To ensure accurate measurement on the 3D printer, the width of the severed leg was measured in addition to its length. Measurements were acquired at the time of admission and submitted to the prosthesis manufacturer in cases when the leg has already been amputated. The prosthesis was ready after around two weeks.

Phase 4—Prototype production: The different prototypes were produced with the 3D printing machine—Creality CR10 V3, using first the material Polylactic Acid (PLA) and then the polyamide/nylon.

Phase 5—Prosthesis placement: The first prototype (P1) was placed using an intramedullary nail that was inserted through the tarsometatarsus perpendicularly and bent downward from both sides. Next, the team inserted the nail into a 20 cm^3^ syringe filled with epoxy glue, allowed it to set for two to three hours, and then removed the fixing bandage.

**Figure 6:**
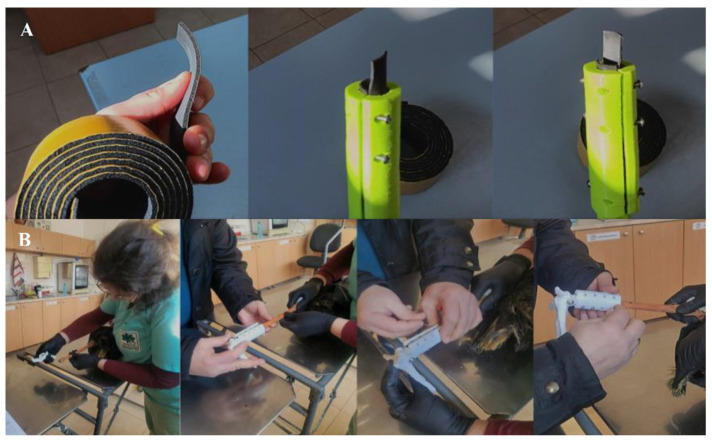
Methodology for the placement of the second and third prototype prostheses. (**A**) Self-adhesive rubber insulation tape. (**B**) Placement of the fitting piece and tightening of the screws using a hex key.

The second and third models have the same application methods. Before placing the prosthesis, a self-adhesive rubber insulation tape is placed between the limb and the prosthesis itself (Figure 6A). Then, the first step is to clean and disinfect the stump; afterwards, the prosthesis fitting piece was placed and the screws were tightened with a hex key (Figure 6B).

**Figure 7:**
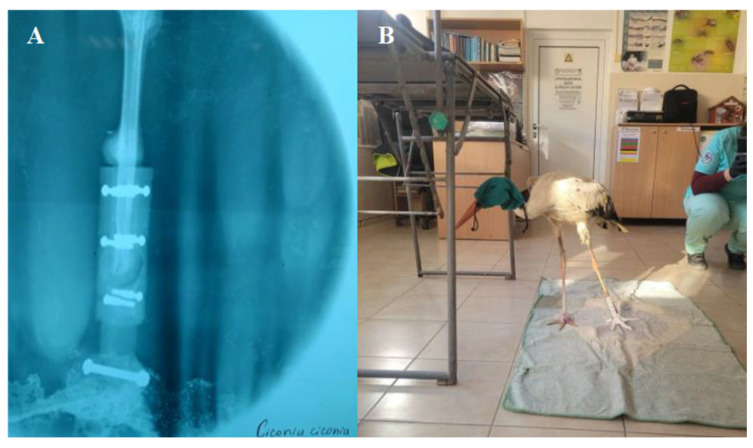
(**A**) Radiograph of the partial limb with the prosthetic device; (**B**) Functionality evaluation and analysis of animal behaviour.

Phases 6 and 7—Functionality evaluation and analysis of animal behaviour: After the placement of the prosthesis, it is necessary to take an x-ray and then evaluate a functionality in the real context of the natural behaviour of the animal. It is the longest and most complex process, as it involves a study of the animal’s behaviour and adaptability. Some basic functions were assessed, such as standing, walking, eating and flying (Figure 7). The prototype must be examined and modified if issues are found to try to better meet the requirements.

## 4. Results

Table 2 provides a summary description of the recovery outcomes of twelve juvenile white storks (*Ciconia ciconia*) admitted to the Green Balkans Wildlife Rehabilitation Center due to various injuries or conditions—for more detailed information consult the Supplementary Materials.

A total of 10 storks were submitted for amputation surgery and the remaining 2 suffer previous traumatic amputation in the wild. All 12 white storks underwent orthopedic prosthetic placement. The causes of admission included different types of bone fractures, amputations or necrosis. The lengths of the amputated segments varied between 1 cm and 13.5 cm.

Three distinct prototypes of orthopedic prostheses were designed and developed. The first one (P1) was placed in six animals and its main disadvantages were the simplistic and anatomically design and the prosthesis fixation method was not ideal, as they eventually detached, causing small wounds at the attachment site, which subsequently required treatment with iodine application. The model P2 was applied in four storks and in one of the birds, it was later replaced with the model P3. The second prototype demonstrated insufficient resistance to impact, leading to three fractures (in three of four individuals) of the prosthesis’s first digit. Additionally, the material’s low friction coefficient rendered the device excessively slippery, compromising functionality. To overcome these limitations, a modified version of prototype 2 was developed and implemented in the stork that was later released into the wild with GPS tracking. In this variation, the first digit of the prosthesis was removed, and methacrylate was applied to the plantar surface to enhance grip and friction. In the subsequent design phase for the P3 model, the first digit was removed entirely, and a more impact-resistant material was selected to improve durability and overall performance. Upon further analysis, it was determined that the rubber tape progressively thinned and lost its initial rigidity, leading to instability and excessive mobility of the prosthesis, so the self-adhesive rubber was replaced with a 2 mm thick insulating rubber tape, which demonstrated greater long-term stability. The model P3 was placed in the last two storks. These prostheses proved effective in supporting the birds’ weight, allowing gradual adaptation. The time required for the storks to adapt to their prostheses ranged from 1 to 5 days. Although some individuals exhibited a residual limp, all resumed normal feeding behaviours after one or two days, and the majority regained functional flight capabilities.

Despite these rehabilitative efforts, the outcomes varied—nine birds stayed at the rehabilitation centre, while three were released into the wild. The birds that achieved full recoveries were released in the vicinity of Opan village, situated approximately 26 km south of Stara Zagora.

One individual, which was fitted with a variation of the P2 model, was also equipped with a GPS transmitter, allowing for the monitoring of its movements over a period post-release. Although prototype 3 had already been developed, it was delivered to the centre shortly before this stork’s release back into the wild. This situation posed a dilemma: if the new prototype were to be tested and the bird released with it, it would require a full adaptation process once again, necessitating an additional winter at the centre. Such an extended period in captivity was deemed undesirable, as it would further limit the animal’s natural behaviours and well-being. Therefore, it was decided to create a variation of model 2 to address its limitations, specifically to prevent the fracture of the first digit by removing it and applying methacrylate to enhance grip and friction with the ground. Survival times varied considerably, ranging from a few months to over three years.

**Figure 8:**
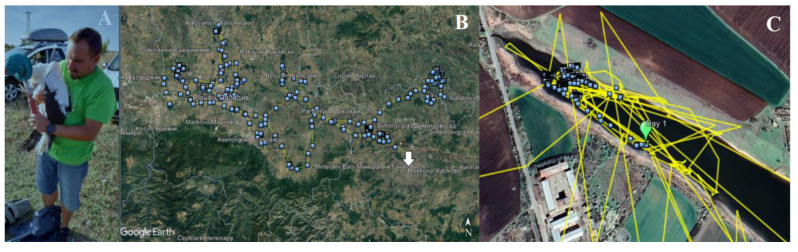
(**A**) Release in the wild of the stork CR97, with the GPS device and a variation of the prototype 2 (**B**) Total of 1060 GPS points recorded (**C**) Initial five days post-release movements.

As previously described, one of the white storks that achieved complete recovery was fitted with a GPS tracker prior to its release into the wild (Figure 8A).

The GPS device used on the stork was the OrniTrack-30 model, a solar-powered GPS-GSM tracker, with no external antennas, weighing 30 g and measuring 61 mm × 25 mm × 23 mm. When fully charged, the battery is capable of logging approximately 1000 positions without requiring additional recharging. Regarding data storage, the device has a capacity of 128 MB, sufficient to store nearly 2,000,000 records.

This allowed for the tracking of its flight patterns and the monitoring of its behaviour while adapting to life with the prosthesis. The stork was released in Opan on 14 September 2023, and GPS data were recorded until 29 September of the same year, covering a period of 16 days. A total of 1060 GPS points were recorded, with a daily average of 65.3 points (Figure 8B). During the initial five days post-release, the stork remained largely within the same area, with movements limited to approximately 300 m (Figure 8C).

From 23 September onward, the stork exhibited a marked increase in its range of movement, initially travelling approximately 11 km from its release point. The final GPS data recorded on 29 September, positioning the bird in the city of Haskovo, located approximately 32 km from the original release point. In total, the distance covered by this bird during the period in which the GPS device recorded data were approximately 470 km.

## 5. Discussion

Many birds admitted to rescue centres suffer severe injuries, often leading to euthanasia due to the impossibility of returning them to the wild. However, in the case of critically endangered species, every individual matters, and interventions like amputation and translocation to zoos or breeding programmes are attempted. According to this view, emerging technologies like orthopedic prostheses offer promising solutions to the improvement of animals’ welfare.

This study explores the use of 3D-printed orthopedic prostheses in wildlife, focusing on white storks (*Ciconia ciconia*), a species commonly admitted to rehabilitation centres across Europe [11]. The main reason for their admission is traumatic injuries [9,12,13,14], typically resulting in amputations. The most groundbreaking aspect of this project involved the reintegration of animals fitted with orthopedic prostheses into their natural habitats. Remarkably, one of these animals was released equipped with GPS tracking technology, enabling the acquisition of data regarding its locomotion and behaviour within its natural environment. A thorough review of the literature revealed no scientific publications documenting a similar case.

The prosthetic models successfully tolerated the weight of the birds, and the adaptation period ranged from 1 to 5 days. All the individuals landed safely and regained normal dietary habits within 1 to 2 days. Some limitations included the lack of pre- and post-implantation radiographic imaging that restricted the assessment of anatomical and functional integration.

Fixation methods also created challenges. Three prototypes were tested: the first used an intramedullary pin, but it failed as the prosthesis eventually detached, causing minor wounds. Additionally, this fixation method carries a high risk of infection, a critical concern in avian orthopedic prosthetics. Some authors recorded severe post-operative infections in a white-naped crane (*Grus vipio*) following the implantation of a titanium prosthesis, which led to euthanasia [15]. Conversely, one case from 2021, that a team were able to successfully implant an osseointegrated prosthesis in a bearded vulture (*Gypaetus barbatus*) with infection prevention through proper wound care and antibiotic therapy [8]. These reports identify infection control as a key aspect of avian prosthesis success.

There is a notable lack of detailed measurements regarding the forces involved in locomotion, both with and without the prosthetic device. These measurements are crucial for evaluating the effectiveness and impact of the intervention and may also help mitigate excessive loading on the non-amputated limb, thereby preventing the onset of pododermatitis. A study from 2018, related a design for a prosthetic foot for a peacock and emphasized the importance of force analysis in the success of the project [16]. Similarly, a team of researchers developed a toucan (*Ramphastos tucanus*) prosthetic foot and demonstrated how asymmetry of weight could lead to chronic ulcers and infections [17]. These findings underscore the need for dynamic prostheses that adapt to different surfaces, allowing birds to balance and execute natural activities without restriction.

One significant limitation of the present study was the short duration of GPS transmissions, which lasted only 15 days. This constraint limited the study of the animal’s long-term survival in the wild and prevented a follow-up assessment to evaluate the potential development of pressure sores. In 2018, a 3D-printed prosthetic leg was developed for a Red-lored Amazon Parrot (*Amazona autumnalis*), which the bird wore comfortably for two months. However, upon removal, a small scab was observed, suggesting a pressure sore and emphasizing the importance of long-term follow-up assessments [18].

Lastly, the material used for their fabrication, which, despite the evolution of the material from the second prototype (PLA) to the third prototype made with polyamide, the material selection has not yet been fully optimized. While PLA experienced some fractures at the level of the first digit, polyamide, although more resistant, still demonstrates limited durability under harsh environmental conditions, like the issues encountered with PLA. This limitation is particularly relevant in the case of the studied species, the white stork, which inhabits humid and wetland ecosystems. In 2018, a study assembled a prosthetic foot using 3D printing technology with fused deposition modelling (FDM) and acrylonitrile butadiene styrene (ABS) as the material [16]. ABS was selected not only for its lightweight properties but also for its rigidity and durability, with an expected lifespan of 50 to 70 years, ensuring resistance to deformation or failure caused by atmospheric conditions over time.

Regarding future improvements in this still underexplored field, the development of orthopedic prosthetics for wild birds could be significantly advanced through the innovative biomimetic approaches, for example, in 2024, a team’ research focused on designing and testing 3D-printed robotic models inspired by bird claw configurations—specifically anisodactyl and zygodactyl structures [19], which are commonly seen in species adapted for perching, hunting, or climbing. These models demonstrated enhanced grasp mechanics due to features such as a smaller, centralized palm structure and curved, talon-like distal digits, which improved their ability to securely grip and support various objects. Applying similar principles to orthopedic prosthetics for wild birds could improve functionality and adaptability, particularly for species requiring the recovery of behaviours like perching or climbing. To address the ecological and biomechanical needs of birds, future developments should also prioritize lightweight and durable materials while integrating advanced testing methods to optimize grasp efficiency and environmental compatibility.

## 6. Conclusions

The development of highly advanced orthopedic prostheses, designed to facilitate optimal locomotion and functional utilization of limbs or beaks and hence overall individual well-being, constitutes a crucial tool in the conservation of endangered species. This approach should be subject to continuous and rigorous investigation to refine prosthetic prototypes and materials, with the overarching objective of creating solutions that are not only anatomically suitable and comfortable for the animals but also economically sustainable and durable. Such innovations are essential for enabling the successful reintegration of these animals into their natural environments, ensuring their survival without compromising their life expectancy in the wild. Nevertheless, the successful achievement of these objectives depends on the investigation of an additional critical factor: the GPS monitoring of animals following their release. This method is indispensable for assessing the adaptation and survival rates of animals utilizing orthopedic prostheses, thus determining the true feasibility of this intervention as a viable and long-term conservation strategy. This pioneering work represents a promising step forward, emphasizing the importance of interdisciplinary collaboration between veterinarians, engineers, and conservationists to refine these technologies to meet the ecological, biomechanical, and economic challenges of wildlife rehabilitation.

## Figures and Tables

**Table 1:** Summary of different prosthetic leg models developed.

Prototype	Year	Material	Method	Advantages	Disadvantages	Weight
P1	2014	Syringe and epoxy resin	Handcrafted	Accessible materials, practical solution	Less durable, not optimized for comfort	-
P2	2021	Polylactic Acid (PLA) *	3D-Printed (Creality CR10 V3)	Inexpensive, biodegradable, easy to print	Low heat and impact resistance, not for outdoors	100 g
P3	2023	Polyamide/Nylon *	3D-Printed (Creality CR10 V3)	More resistant and lighter, improved comfort	Not suitable for humid environments, prone to warping	80

* Both P2 and P3 contain rubber inside for additional support and comfort.

**Table 2:** Summary of the recovery outcomes of juvenile white storks (*Ciconia ciconia)* admitted to the Green Balkans Wildlife Rehabilitation Center.

Parameter	Value
Number of animals	12
Weight	1.73–2.86 kg (mean = 2.31 kg, N = 12)
Dates of admission	11 August 2014–28 July 2023
Amputation or surgery?	2 already amputated, 10 surgically amputated
Missing leg length	1–13.5 cm (mean = 4.25 cm, N = 12)
Bottom end diameter	1–2.3 cm (mean ≈ 1.42 cm, N = 12)
Prosthetic models	P1 (6), P2 (3), P3 (3)
Prosthetic material	Syringe and epoxy resin (6), PLA (3), Polyamide (3)
Time until prosthesis support	1–5 days (mean ≈ 2.83 days, N = 12)
Walking with a limp?	11 Yes, 1 No
Eating after prosthesis?	12 Yes; 1–2 days (mean ≈ 1.17 days, N = 12)
Flying?	11 Yes, 1 No
Using prosthesis for landing?	10 Yes, 2 No
Release status	3 released (Opan, Stara Zagora)
GPS Tracking	1 Yes; 2 No
Survival time	0.34–3.77 years (mean ≈ 1.83 years, N = 8)

## Data Availability

The original contributions presented in this study are included in the article. Further inquiries can be directed to the corresponding author.

## Supplementary Materials

The following supporting information can be downloaded at: https://www.mdpi.com/article/10.3390/biology14030265/s1, Table S1: Detailed description of the recovery outcomes of juvenile white storks (*Ciconia ciconia*) admitted to the Green Balkans Wildlife Rehabilitation Centre.
