# Supplementary material for: Assessing the Use of 3D-Model Prostheses in White Storks: A Promising Method in Rehabilitation of Injured Wildlife
Source: Biology (Basel). 2025 Mar 5;14(3):265. doi: 10.3390/biology14030265 (PMC11940723; doi:10.3390/biology14030265)
Supplement: Supplementary file 1 [file biology-14-00265-s001.zip › biology-3444359-supplementary.pdf]

Supplementary Materials

Table S1. Detailed description of the recovery outcomes of juvenile white storks (*Ciconia ciconia)* admitted to the Green Balkans Wildlife Rehabilitation Center

**Number**	**1**	**2**	**3**	**4**	**5**	**6**	**7**	**8**	**9**	**10**	**11**	**12**
**Stork ID**	Z-82	H - 92	H-17	B0646/49	Red 79	H-64	7-09510	CR 39	CR97	CR 70	CR56	CR79
**Age**	Juvenile	Juvenile	Juvenile	Juvenile	Juvenile	Juvenile	Juvenile	Juvenile	Juvenile	Juvenile	Juvenile	Juvenile
**Weight**	2,86	1,84	2,68	2,5	1,73	2,29	2,49	2,43	2,62	2,37	1,74	2,16
**Date of admission**	11/08/2014	06/09/2014	06/07/2015	01/08/2015	07/07/2016	26/07/2016	28/07/2021	01/07/2022	15/07/2022	22/07/2022	12/06/2023	28/07/2023
**Cause of admission**	Fracture of the metatarsus	Fractured beak; Partial foot amputation	Traumatic foot amputation	Leg necrosis	Exhaustion; Consolidated fracture of the tarsometatarsus	Dry necrosis of the plantar surface of the foot	Laceration in metatarsus	Necrosis of the foot	Necrosis of metatarsus	Necrosis of foot; luxation in the hip joint	Fracture of the left wing - radius and ulna; Fracture leg	Severely contracted achilles tendon on leg
**Amputation or surgery?**	Amputation surgery	Already amputated	Already amputated	Amputation surgery	Amputation surgery	Amputation surgery	Amputation surgery	Amputation surgery	Amputation surgery	Amputation surgery	Amputation surgery	Amputation surgery
**Foot**	Right	Left	Right	Right	Left	Left	Right	Right	Left	Left	Right	Right
**Missing length (cm)**	4	4	2	3	3	2	5,5	1	3	4	6	13,5
**Bottom End Diameter (cm)**	1,5	1	1	1	1,5	1	1	1,5	1	1,5	2	2,3
**Prosthetic model**	Syringe and epoxy resin	Syringe and epoxy resin	Syringe and epoxy resin	Syringe and epoxy resin	Syringe and epoxy resin	Syringe and epoxy resin	Red/PLA - replaced with Green/Polyamide in 09/2023	Red/PLA	Red/PLA replaced with Red/PLA Variation	White/PLA - replaced with red/PLA	Green/ Polyamide	Green/ Polyamide
**Does it support the weight of the prosthesis?**	Yes	Yes	Yes	Yes	Yes	Yes	Yes	Yes	Yes	Yes	Yes	Yes
**How long after the prosthesis?**	2 days	2 days	4 days	3 days	3 days	3 days	5 days	3 days	4 days	5 days	3 days	1 day
**Does he walk with a limp?**	Yes	Yes	Yes	Yes	Yes	Yes	Yes	Yes	No	Yes	Yes	Yes
**Does it eat?**	Yes	Yes	Yes	Yes	Yes	Yes	Yes	Yes	Yes	Yes	Yes	Yes
**How long after the prosthesis?**	2 days	1 day	1 day	1 day	1 day	1 day	2 days	1 day	1 day	1 day	1 day	1 day
**Does it fly?**	Yes	Yes	Yes	Yes	Yes	Yes	Yes	Yes	Yes	Yes	No	Yes
**Can it use the prosthesis to land?**	Yes	Yes	Yes	Yes	Yes	Yes	Yes	Yes	Yes	Yes	No	No
**Date of release**	Stayed at the rescue centre	Stayed at the rescue centre	Stayed at the rescue centre	03/10/2015	Stayed at the rescue centre	Stayed at the rescue centre	Still at the rescue centre	Stayed at the rescue centre	14/09/2023	30/03/2023	Stayed at the rescue centre	Stayed at the rescue centre
**Location of release**	-	-	-	Opan, Stara Zagora	-	-	-	-	Opan, Stara Zagora	Opan, Stara Zagora	-	-
**Body found (yes/no)**	-	-	-	No	-	-	-	-	No	No	-	-
**Location/GPS**	-	-	-	No	-	-	-	-	Yes	No	-	-
**Date of death**	19/05/2018	11/07/2015	30/09/2018	-	15/07/2018	15/09/2019	-	08/03/2023	-	-	10/01/2024	29/11/2023
**Autopsy (yes/no)**	Yes	Yes	Yes	-	Yes	Yes	-	Yes	-	-	Yes	Yes
**Cause of death/ Necropsy findings**	Liver dystrophy	Enterocolitis	Kidney dystrophy; heart attack	-	Exhaustion	Hepatomegaly	-	Tricuspid valve hypertrophy	-	-	Exhaustion	Intestinal obstruction
**Post-surgery survival (year)**	3.77 years	0.84 years	3.24 years	-	2.02 years	3.14 years	-	0.68 years	-	-	0.58 years	0.34 years
